# Optical measurement methods of the 3D-position stability of implant-abutment connections - an in vitro study

**DOI:** 10.1186/s12903-025-06265-y

**Published:** 2025-06-06

**Authors:** S. Wenger, L. Martin, A. Kernen-Gintaute, C. Müller, K. Nelson, Florian Kernen

**Affiliations:** 1 Department of Oral and Maxillofacial Surgery, Translational Implantology, Medical Center, Faculty of Medicine, University of Freiburg, Hugstetterstraße 55, 79106 Freiburg, Germany; 2 Faculty Mechanical and Medical Engineering, Furtwangen University, Furtwangen, Germany; 3 Department of Prosthetic Dentistry, Medical Center, Faculty of Medicine, University of Freiburg, Freiburg, Germany; 4 Department of Microsystems, IMTEK, University of Freiburg, Freiburg, Germany

**Keywords:** Implant-abutment connection, Coordinate measuring machine (CMM), Vertical and horizontal deviation, 3D optical measurement, Dental implant

## Abstract

**Introduction:**

For ideal occlusion and passive fit of implant-retained prosthetic restorations minimal tolerance of the position of the abutment in the implant after dis- and reassembly is essential. Methods to examine the three-dimensional (3D) positional stability of implant-abutment connections (IAC) vary and their accuracy and applicability have not been assessed. The aim of this study was to evaluate non-contact optical measurement devices for 3D-feasibility, accuracy, repeatability, time efficiency and complexity of use.

**Methods:**

Five devices capable of contactless, optical methods (Profile Projector; Digital Image Correlation (DIC); Profilometer; Confocal Technology; Coordinate Measuring Machine (CMM)) were examined regarding their 3D-feasibility, accuracy, repeatability, time efficiency and complexity. The parameters were quantified and scored using a decision matrix. In an experimental set-up the method (device) with the highest score was used to measure the position stability as follows: four implants with a butt joint (BJ) and a conical (CON) IAC were embedded in an aluminum block and dis- and reassembled ten times with the abutment screw being hand-tightened (∑1–5) and torque-tightened (∑6–10).

**Results:**

The CMM achieved the highest score in the decision matrix with 40/50 points (range: 10–50 points; 10 requirements not met, 50: requirements fully met) while the Profile Projector, the DIC, the Profilometer and the Confocal Technology achieved 30, 32, 34, 38 points, respectively. Using the CMM the mean rotational freedom in BJ vs. CON was 0.32° ± 0.16° vs. 0.21° ± 0.25° (hand-tightened) and 0.36° ± 0.09° vs. 0.20° ± 0.22° (ratchet), respectively. The maximum vertical deviation of the abutment position after re-assembly was 7.2 μm ± 2.1 μm (BJ) and 24.4 μm ± 2.1 μm (CON).

**Conclusion:**

The data acquired suggest that the CMM with its non-contact, optical measurement method is the most appropriate to investigate the 3D positional stability of the IAC in different implant systems. As previously described distinct differences between BJ and CON IACs were found. CON connections exhibit a higher vertical deviation when the system specific torque value was applied.

## Background

The accuracy of the mating zone between implant and abutment, the implant-abutment connection (IAC), is of great importance in clinical practice. Dis- and reassembly of the implant components during the fabrication of the superstructure by the dental technician and dentist is required with highest positional stability to ensure a proper occlusion and a passive fit of the implant-supported restoration [1]. Passive fit is essential to prevent mechanical complications, e.g. ceramic chipping, implant fractures or screw loosening [2].

The majority of the IACs in two-piece implants show an internal anti-rotational index based on either cam-groove or polygonal positional (hexagon, octagon, torx) designs and have progressively replaced external hexagonal butt joint IACs [3]. Cam-groove connections are characterized by the lowest freedom of rotation, since the implant wall crosses the rotation path almost orthogonally with a maximum rotation of 1.4° compared to other internal and external IACs [4, 5]. Today, morse taper connections, also known as conical connections, and butt joint IACs are available [6]. Conical connections are usually based on a press-fit characterized by an axial displacement of the abutment into the implant when tightening the connecting screw with a defined torque. Butt joint connections join (vertical stop) on a horizontal or slightly angled surface between the implant and the abutment; the preload of the screw defines the loading capacity of the connection [7, 8].

Although the 3D positional stability of IACs is a very important factor in clinical practice, information about the precision of current IACs is scarce, and varying measurement methods provide deficient information for comparison [9–11]. Measuring the mechanical behavior and dimensions of dental devices is complex due to varying shapes necessitating non-contact measurement methods. Different devices capable of non-contact, optical measuring methods have been introduced in literature with diverging areas of application and limitations, e.g. Profile Projectors, Digital Image Correlations (DIC), Profilometers, Confocal Technology and Coordinate Measuring Machines (CMM) [12–19].

A review of the existing literature revealed limited emphasis on the selection and justification of measurement technologies for evaluating IACs. While numerous studies have investigated IACs and their dimensional offset, the rationale behind the choice of measurement devices is rarely discussed in detail.

Measurements with the Profile Projector are based on telecentric objectives. Silhouettes by illuminating objects with axis-parallel rays (since its lenses have their entrance or exit pupil, or both, at infinity) are created. It is mostly used in diameter and thickness measurements on precision workpieces [20]. The DIC provides a camera-based measuring method, whereby it is capable of registering and recording movements of surface structures with cameras. For image correlation, digital images are stored to measure the displacement field within the region of interest based on a greyscale similarity. It is mainly used in deformation measurements (e.g. shrinkage behavior in dental composites) and mechanical behavior evaluations in dental restorations [21–23]. The Profilometer is mostly used to determine surface roughness in dental materials. Measurements are performed based on an optical light band that emits light strip patterns that reflect the surface of test elements [24]. A receiver processes the deflection via triangulation. The device works with micro and macro settings to achieve different accuracies. However, the field of view shrinks with increasing precision [25]. Measurements with the Confocal Technology are based on a confocal microscope, which means that measurements to capture the topography, layer thickness and roughness of the surfaces of test elements and to generate razor-sharp images in nanometer range via magnification are possible [26]. Finally, measurements with the CMM are based on a reference coordinate system, therefore, spatial coordinates of points on a workpiece are recorded. Measurements can be performed with tactile or optical technology. The CMM is able to generate contours and to calculate metrologically relevant elements which, depending on light intensity, form a pixel matrix that is converted into an electrical potential. Based on the electrical potential, a gray value (0-255) is assigned to the pixel matrix [27]. The data is evaluated based on a python script.

Due to the diverging possibilities, the aim of this study was to evaluate non-contact optical measurement devices for 3D-feasibility, accuracy, repeatability, time efficiency and complexity of use. It is intended to serve as a basis for further and repeatable IAC testing. The null hypothesis (N0) of this study is that there are no significant differences in vertical and rotational between conical and butt joint IACs measured with a non-contact optical experimental setup.

## Materials and methods

### Measuring accuracy

The measurement accuracy was set at 2 μm and 0.1° as based on previous studies [4, 5, 10, 11, 15, 28–34]. Relative measurements were carried out, making the systematic error irrelevant.

## Measuring device

In collaboration with the Department of Microsystems (IMTEK) measuring devices and methods, to which we had access (in-house, IMTEK or nearby institutes) were supervised. Five established devices capable of contactless, optical measurement methods were examined (Profile Projector, DIC, Profilometer, Confocal Technology and the CMM). Feasibility tests were carried out. Therefore, an abutment was hand-tightened onto an implant and the IAC was visualized and measured.

## Decision matrix

**Fig. 1:**
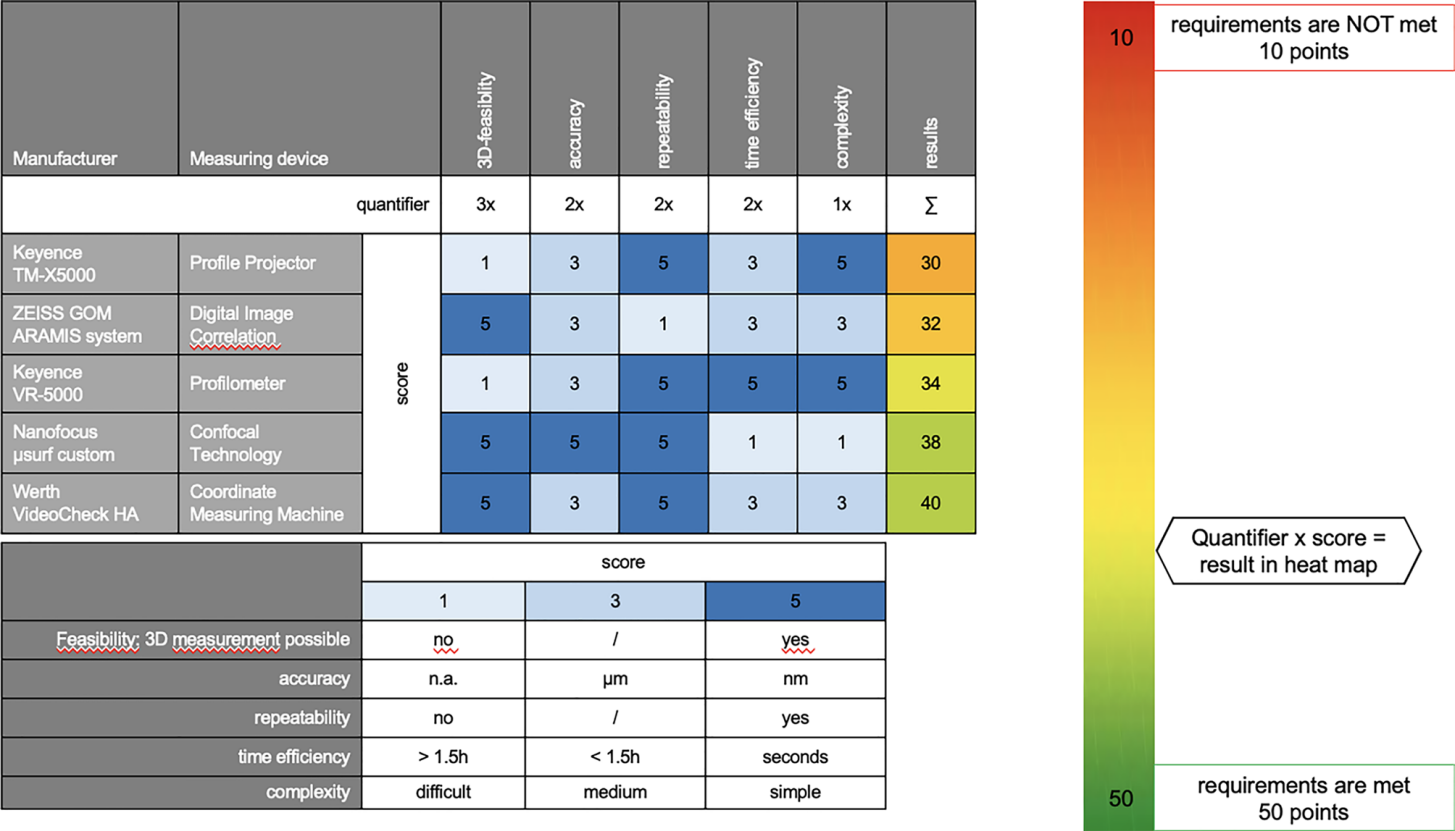
Decision matrix for evaluating the most suitable measurement device

Evaluation for the most suitable measuring device was standardized using a decision matrix as previously described [35]. Requirements (3D-feasibility, accuracy, repeatability, time efficiency and complexity) were defined and scored based on its importance in this proof of concept as visualized in Fig. 1. 3D-feasibility was rated with 3 points. Accuracy was quantified with 2, repeatability with 2, time efficiency with 2 and complexity with 1 point, respectively. The 3D-feasibility was a yes / no decision (5 / 1 points). The accuracy was scored from 1-3-5 with increasing precision from not applicable (n.a.), µm to nm, whereas the repeatability was a yes / no decision (5 / 1). Repeatability is the variation that is observed when the same operator measures the same part many times, using the same gauge, under the same conditions [36]. The time expenditure was rated from > 1,5 h (h) to < 1,5 h to seconds (1-3-5). There is no universally accepted definition of complexity in literature, since complex systems are influenced by various interrelated factors. In this context, we defined the complexity of a measuring machine based on the technical knowledge required for experimental setup, execution, and data analysis (simple: 5, medium: 3, difficult: 1) by the operator. “Simple” means that preparations, measurements and follow-up can be carried out with little prior knowledge by the operator. Medium complexity requires prior knowledge to analyze the data, while the experimental setup and execution can be performed with little technical knowledge. A measurement device is complex if experimental setup, execution and data analysis obliges prior knowledge by the operator. All measuring devices were quantified with the quantifier and score multiplied for the overall ranking (10–50 points; 10 points: requirements are not met, 50 points: requirements are completely met).

## Experimental setup

**Fig. 2:**
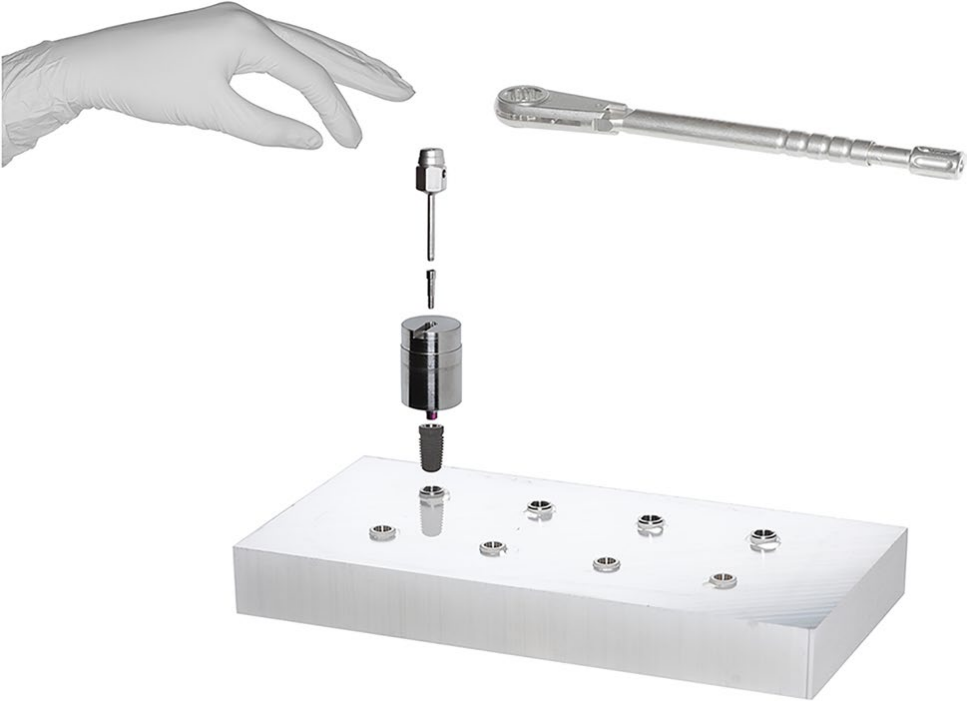
Measurement setup consisting of aluminum base, two implant systems (BJ (top) and CON (bottom)), abutment, abutment screw, all tightened by hand or screw driver and ratchet

A 5 × 10 cm ALPLAN^®^ aluminum block formed the basis of the final experimental setup. Its top surface was unmodified whereas its sides were eroded for a sharper design of the edges. Eight holes were drilled to fit the outer diameter and length of the implants. A drainage channel was added at the base for drainage of excess adhesive. The implants were embedded 3 mm under the IAC with a methyl methacrylate adhesive (X60, HBM Germany), which was placed into the cavities with a syringe, taking care not to spread the adhesive over the implant shoulder. All implants were aligned based on the cam-groove in the same direction. Four CAMLOG^®^ SCREW-LINE Promote^®^ Plus (4.3 × 11 mm) (BJ) and four CONELOG^®^ SCREW-LINE Promote^®^ Plus (4.3 × 11 mm) (CON) implants were examined. The blanks (PreFace^®^ abutments, 12 × 20 mm, Medentika) were processed with an Ultra Precision Machine (UPM) and adjusted by hand (assembly 1–5) or screwdriver and torque ratchet (assembly 6–10), as visualized in Fig. 2.

## Final measurements of IAC

**Fig. 3:**
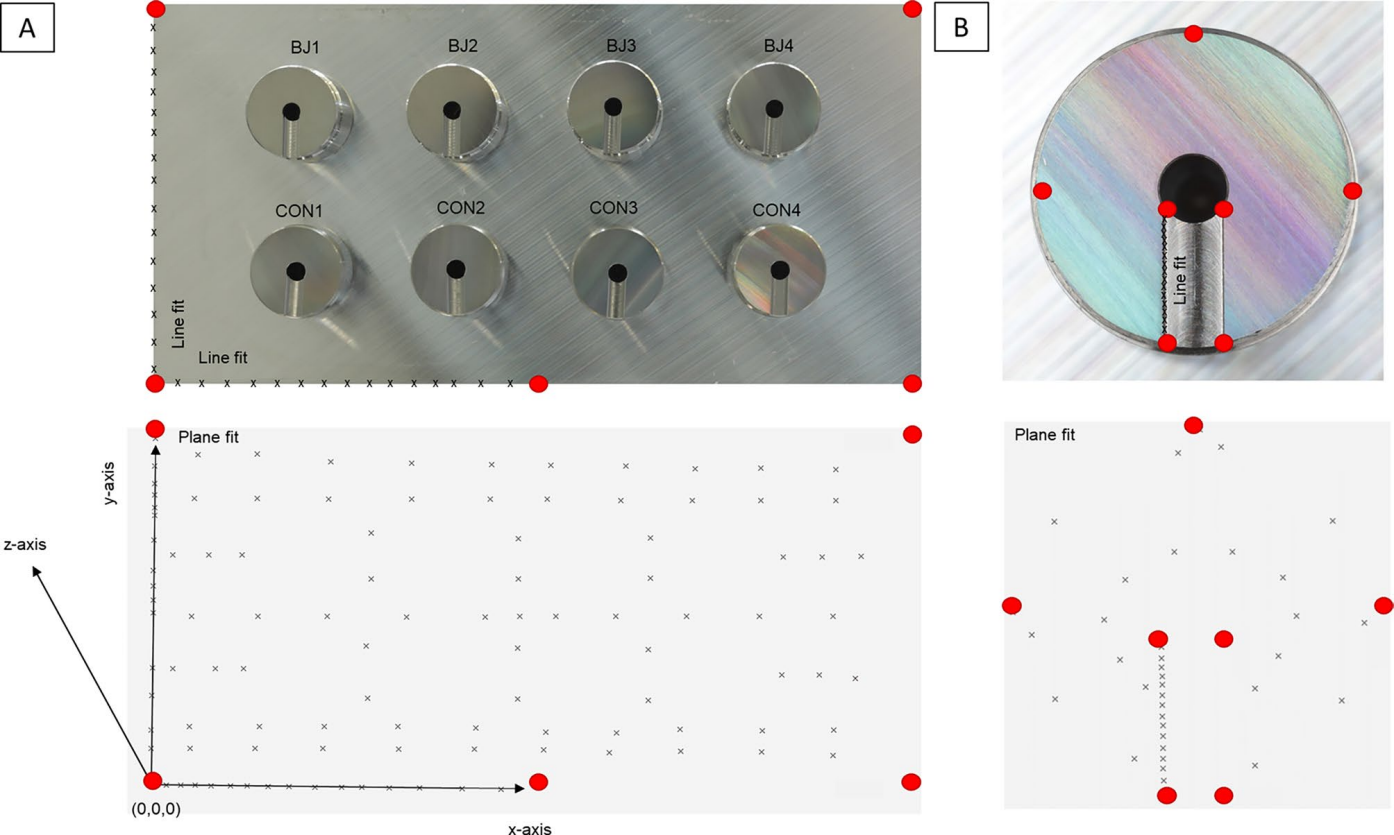
Definition of the 3D coordinate system to align the measurement setup in the CMM. (**A**) Top: View of the measurement setup with illustration of the reference points in red to localize the sample in the CMM. 15 points on x- and y-axis, respectively, are used for line fit. Bottom: Schematic visualization of reference coordinate system. 75 points are used to define plane fit on the aluminum base surface. (**B**) Abutment surface with reference points in red, line fit (15 points) and plane fit (20 points)

Measurements were conducted using an optical system (600 × 600 × 350 mm field of view) with the CMM placed in a temperature-controlled environment (20 °C) to minimize thermal fluctuations. To reduce vibration effects, the CMM was mounted on a vibration-dampening table. Autofocus was used to standardize magnification and lighting. In order to measure the IACs with the CMM, a reference coordinate system had to be defined. A non-linear plane fit and a linear line fit was used to determine how many points were necessary to achieve accuracy in vertical deviation and rotation. All points were precisely positioned manually in all assemblies 1–10 and measurements were performed with autofocus (Fig. 3). The vertical deviation was measured for each abutment based on the deviation on the z-axes of the plane fits, relatively to the first measurement. For the rotational deviation (x/y-axes) straights of the occlusal marking of the abutments (line fit) were assumed and their deviations of each other were compared as visualized in Fig. 3B.

### Statistical analysis

Descriptive analysis of the scores were performed. The vertical deviation and rotation were evaluated using a Python Script. Statistical analysis (mixed linear models) was performed with STATA (Version 17.0, StataCorp, CollegeStation, Texas USA) using box plots to compare vertical and rotational deviation in BJ 1, 2, 3, 4 vs. CON 1, 2, 3, 4 for each assembly (adjusted by hand ∑ 1–5 or screwdriver and torque ratchet ∑ 6–10). Additionally, the measured values were summed and compared (BJ 1–4 vs. CON 1–4 ∑ 1–10).

## Results

### Evaluation of the measuring device

The CMM (Werth VideoCheck^®^ HA) accomplished the highest score (range of 10–50 points in decision matrix: 10 points = requirements are not met; 50 points = requirements are completely met) with 40/50 points in the decision matrix compared to the other devices with 30, 32, 34 and 38 points in the Profile Projector, DIC, Profilometer and Confocal Technology, respectively. The 3D-feasibility was possible, high accuracy (µm) was achieved in CMM and the measurements repeatable, although the reference coordinate system had to be newly defined with each assembly. Time required per assembly was < 1.5 h and medium complexity remained.

Although the vertical deviation of IACs could be determined with the Profile Projector, the rotational deviation of IACs could not be examined due to the 2D representation of the silhouettes (no 3D-feasibility). The accuracy in DIC, although it is a precise device, is influenced since no standard calibration process of the DIC system is available [37]. Since the aim of this proof of concept was to compare implant systems, we particularly weighted the accuracy and repeatability. The DIC did not meet our requirements due to its lack of calibration and was therefore not further pursued. Since the Profilometer works with macro and micro settings to achieve different accuracies, the field of view (max. 7.6 × 5.7 mm at 40x magnification, min. 1.9 × 1.4 mm at 160x magnification in micro mode) shrunk with increasing precision and therefore only small sections of the IAC could be imaged with sufficient accuracy. The rotational deviation could not be displayed within this window. The reflection of the titanium surface also made measurements impossible, so 3D feasibility was impossible within micro mode. The Confocal Technology works with very small windows and is extremely precise, but measurement was exceptionally time-consuming and complex due to lack of automation.

### Reference coordinate system

Since the CMM is based on a reference coordinate system, an Origin Point (0,0,0) on the x-, y- and z-axes was required. The metal base served as a reference system. The x-axis was defined as the long side of the metal block, y-axis as the short side and z-axis as the respective height of each abutment, respectively, as shown in Fig. 3.

### Theoretical considerations with the CMM

For the non-linear plane fit and the linear line fit, on which the vertical deviation and rotation are based, the total number of measuring points to achieve acceptable error margins had to be determined via statistical analysis (Origin). Under magnification, the edges of the unmodified metal block where partially broken off thus our measuring accuracy of 2 μm and 0.1° was not reached even with a series of measurements of up to 75 measuring points in the line fit. Therefore, the edges of the metal block were eroded while the surface was left unmodified. Subsequently, 15 measuring points were distributed equally over the length of the edges of the metal base of 5 cm, whereby an accuracy of 0.1° was achieved. For the plane fit, 75 points were distributed equally over the surface of the metal base to achieve a height accuracy of 2.1 μm (Fig. 3A). The unmodified surface of the abutments was likewise initially not sufficient to meet the measurement accuracy. Different surface preparation processes were tested including grounding by hand and machine, eroding and processing with an ultra-precision machine (UPM). The measurement accuracy was tested for each surface preparation of abutment at a certain number of measuring points, whereas the modification with an UPM achieved the most accurate results. A linear regression with 15 measuring points was used to determine the freedom of rotation (x/y-axes) resulting in a standard error of 0.14°. The height difference (deviation on z-axis) was calculated using plane fits with 20 measuring points leading to a standard error of 2.1 μm (Fig. 3B). The following applies for the vertical deviation (z-axis): The height (plane fit) of the abutments (BJ 1, 2, 3, 4 ∑1–5, BJ 1, 2, 3, 4 ∑6–10, BJ 1, 2, 3, 4 ∑1–10, CON 1, 2, 3, 4 ∑1–5, CON 1, 2, 3, 4 ∑6–10, CON 1, 2, 3, 4 ∑1–10) were compared relative to each other. For the rotational deviation (x/y-axes) a straight of the occlusal marking of the abutment (line fit) was assumed and their deviations of each other (BJ 1, 2, 3, 4 ∑1–5, BJ 1, 2, 3, 4 ∑6–10, BJ 1, 2, 3, 4 ∑1–10, CON 1, 2, 3, 4 ∑1–5, CON 1, 2, 3, 4 ∑6–10, CON 1, 2, 3, 4 ∑1–10) were determined as shown in Table 1.

**Table 1:** Vertical deviation in BJ and CON (BJ 1, 2, 3, 4 ∑1–5, BJ 1, 2, 3, 4 ∑6–10, BJ 1, 2, 3, 4 ∑1–10, CON 1, 2, 3, 4 ∑1–5, CON 1, 2, 3, 4 ∑6–10, CON 1, 2, 3, 4 ∑1–10) and rotational deviation in BJ and CON (BJ 1, 2, 3, 4 ∑1–5, BJ 1, 2, 3, 4 ∑6–10, BJ 1, 2, 3, 4 ∑1–10, CON 1, 2, 3, 4 ∑1–5, CON 1, 2, 3, 4 ∑6–10, CON 1, 2, 3, 4 ∑1–10)

	BJ1	BJ2	BJ3	BJ4	CON1	CON2	CON3	CON4
**Assembly**	Measurement results [µm] Measuring uncertainty ± 2.1 μm in vertical deviation (z-axis)							
**1**	0	0	0	0	0	0	0	0
**2**	1.3	0.5	0.4	0.8	2.0	4.3	4.3	5.1
**3**	0.5	0.6	0	1.1	5.1	6.8	6.4	8.7
**4**	3.6	3.7	3.3	2.9	6.	6.9	9.0	8.3
**5**	2.1	2.0	3.1	2.8	2.9	3.7	4.0	6.8
**6**	3.6	3.9	3.8	3.0	-12.8	-8.8	-4.1	-9.5
**7**	-2.5	-2.9	-3.0	-4.2	-16.2	-13.8	-10	-15.7
**8**	1.2	1.0	0.7	0.7	-8.8	-9.0	-6.9	-6.8
**9**	1.6	0.8	2.1	1.2	-9.3	-6.7	-4.3	-5.9
**10**	2.4	1.7	2.1	1.3	-8.9	-9.1	-6.3	-4.5
**max. deviation** **∑ 1–5**	**3.6**	**3.7**	**3.3**	**2.9**	**6.7**	**6.8**	**8.9**	**8.9**
**max. deviation** **∑ 6–10**	**6.1**	**6.8**	**6.8**	**7.2**	**7.5**	**7.1**	**5.9**	**11.2**
**max. deviation** **∑ 1–10**	**6.1**	**6.8**	**6.8**	**7.2**	**22.9**	**20.6**	**19**	**24.4**
**Assembly**	Measurement results [°] Measuring uncertainty ± 0.14° in rotational deviation (x/y-axes)							
**1**	0	0	0	0	0	0	0	0
**2**	0.12	-0.06	-0.08	0.02	-0.29	-0.18	0.07	-0.24
**3**	1.16	0.17	0.87	1.03	0.30	0.73	1.12	1.11
**4**	0.92	0.63	0.22	0.54	-0.05	-0.27	0.98	-0.22
**5**	0.41	0.18	0.05	0.18	0.43	0.06	0.72	-0.14
**6**	-0.13	0.39	-0.15	0.27	-0.30	-0.19	0.11	-0.21
**7**	0.12	0.27	-0.04	0.05	-0.47	-0.42	-0.20	-0.15
**8**	0.62	0.38	0.66	0.73	0.42	0.57	0.87	0.83
**9**	0.35	0.10	0.02	0.48	-0.10	0.42	0.61	0.38
**10**	0.67	0.10	0.02	0.48	-0.10	0.42	0.61	0.38
**max. deviation** **∑ 1–5**	**1.16**	**0.69**	**0.96**	**1.03**	**0.72**	**1.00**	**1.19**	**1.34**
**max. deviation** **∑ 6–10**	**0.81**	**0.75**	**0.85**	**0.72**	**0.89**	**1.00**	**1.07**	**1.03**
**max. deviation** **∑ 1–10**	**1.29**	**0.92**	**1.02**	**1.03**	**0.91**	**1.15**	**1.31**	**1.34**

### Vertical deviation

All vertical calculations were performed respective to the initial assembly (hand-tightened). The abutments were inserted 5 times by hand-tightening the abutment screw and 5 times using a a torque ratchet with a torque of 20 Ncm according to the manufacturer´s instructions and its height deviation (z-axis) based on the plane fits were determined. All results were visualized in Table 1. The vertical deviation varied in BJ 1–4 and CON 1–4. The largest maximum vertical deviation (BJ 1–4, ∑ 1–5) was 3.7 μm ± 2.1 μm in BJ 2 and the smallest 2.9 μm ± 2.1 μm in BJ 4, respectively. As for CON 1–4, ∑ 1–5 the largest maximum vertical deviation was 8.9 μm ± 2.1 μm in CON 3 and CON 4 and the smallest 6.7 μm ± 2.1 μm in CON 1, respectively. The largest maximum vertical deviation (BJ 1–4 ∑ 6–10) was 7.2 μm ± 2.1 μm in BJ 4 and the smallest 6.1 μm ± 2.1 μm in BJ 1, respectively. As for CON 1–4, ∑ 6–10, the largest maximum vertical deviation was 11.2 μm ± 2.1 μm in CON 4 and the smallest 5.9 μm ± 2.1 μm in CON 3, respectively. The largest maximum vertical deviation in BJ 1–4, ∑ 1–10 was 7.2 μm ± 2.1 μm for BJ 4 and 24.4 μm ± 2.1 μm in CON 1–4, ∑ 1–10, respectively.

### Rotational deviation

All rotational values were calculated respective to the initial assembly and are visualized in Table 1. The largest maximum rotational deviation in BJ 1–4, ∑ 1–5 was found in BJ 1 with 1.16° ± 0.14° and in CON 1–4, ∑ 1–5 with 1.34° ± 0.14° (CON 4), respectively. The smallest maximum rotational deviation in BJ 1–4, ∑ 1–5 was found in BJ 2 with 0.69° ± 0.14° and in CON 1–4, ∑ 1–5 with 0.72° ± 0.14° (CON 1), respectively. Tightened by screw driver or torque ratchet, the largest maximum deviation was in BJ 1–4, ∑ 6–10 to 0.85° ± 0.14° (BJ 3) and 1.07° ± 0.14° (CON 3) in CON 1–4, ∑ 6–10, respectively. The smallest maximum deviation in BJ 1–4, ∑ 6–10 was found in BJ 4 with 0.72° ± 0.14° and 0.89° ± 0.14° in CON 1 (CON1-4, ∑ 6–10). The largest maximum deviation for all assemblies was 1.34° ± 0.14° in CON4 and the smallest in CON 1 with 0.91° ± 0.14°, respectively.

### Box plots

**Fig. 4:**
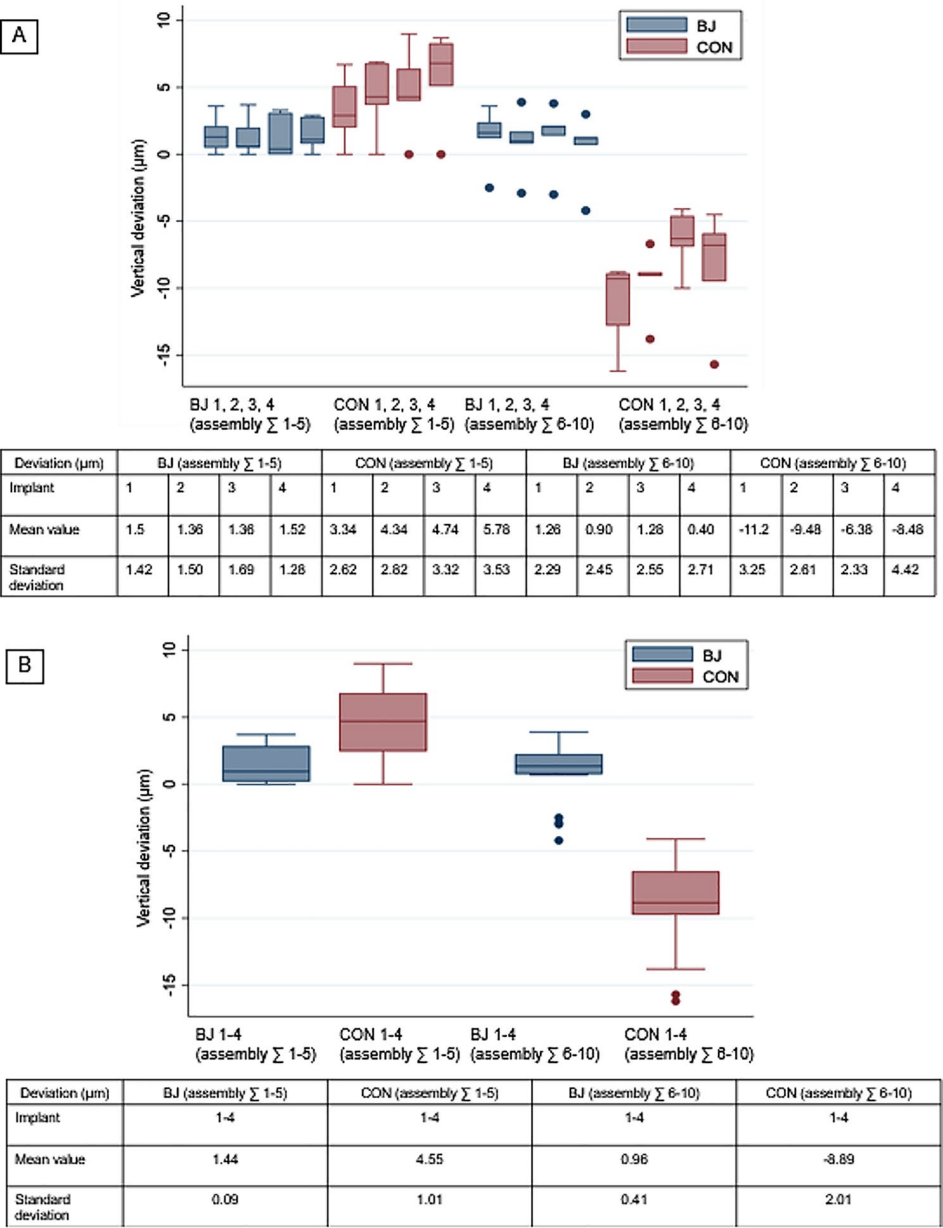
Box plots. (**A**) Vertical deviation in BJ 1, 2, 3, 4 and CON 1, 2, 3, 4 in assembly ∑ 1–5 (hand-tightened) and ∑ 6–10 (tightened by screw driver and ratchet). (**B**) Vertical deviation in BJ 1–4 and CON 1–4 ∑ 1–10

**Fig. 5:**
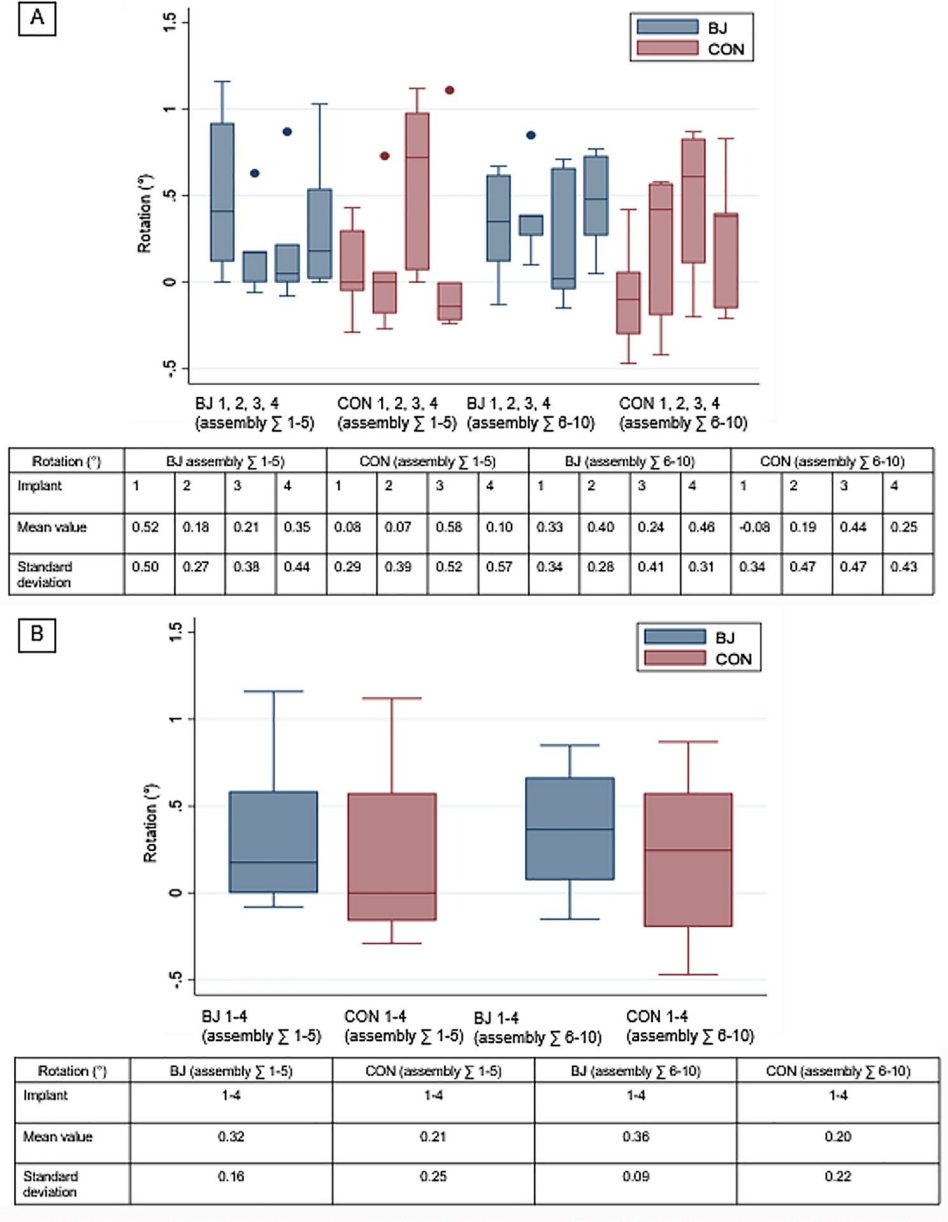
Box Plots. (**A**) Rotational deviation in BJ 1, 2, 3, 4 and CON 1, 2, 3, 4 in assembly ∑ 1–5 (hand-tightened) and ∑ 6–10 (tightened by screw driver and ratchet). (**B**) Rotational deviation in BJ 1–4 and CON 1–4 ∑ 1–10

The box plots in Figs. 4 and 5 illustrate the summarized measurements of BJ and CON, categorized into two conditions: hand-tightened and tightened with a torque ratchet. With hand-tightening, the mean values for BJ 1, 2, 3, 4 ∑ 1–5 ranged from 1.36 μm ± 1.50 μm to 1.52 μm ± 1.28 μm, while for CON 1, 2, 3, 4 ∑ 1–5, they ranged from 3.34 μm ± 2.62 μm to 5.78 μm ± 3.53 μm, respectively. When tightened with a torque ratchet, the mean values for BJ 1, 2, 3, 4 ∑ 6–10 ranged from 0.40 μm ± 2.71 μm to 1.28 μm ± 2.55 μm. For CON 1, 2, 3, 4 ∑ 6–10, the mean values ranged from − 6.38 μm ± 2.33 μm to -11.2 μm ± 3.25 μm, indicating a significant vertical deviation in conical IACs when tightening with a torque ratchet (Fig. 4A). For BJ 1–4 ∑ 1–5, the mean values varied between 1.44 μm ± 0.09 μm and 4.55 μm ± 1.01 μm in CON 1–4 ∑ 1–5. In BJ 1–4 ∑ 6–10, the mean values ranged from 0.96 μm ± 0.41 μm and − 8.89 μm ± 2.01 μm in CON 1–4 ∑ 6–10, respectively. It is evident from Fig. 4B that there is a distinct downward vertical offset in conical IACs. In contrast, Fig. 5A and B show that the type of fixation does not affect rotational deviation, regardless of the IAC type (BJ or CON). For rotational deviation, the mean in BJ 1, 2, 3, 4 tightened by hand ranged from 0.18° ± 0.27° to 0.52° ± 0.50°, compared to a range of 0.07° ± 0.39° to 0.58° ± 0.52° for CON 1, 2, 3, 4. When tightened with a ratchet, the mean rotational deviation for BJ 1, 2, 3, 4 ranged from 0.24° ± 0.41° to 0.46° ± 0.31°, whereas for CON1, 2, 3, 4, it increased to a range of -0.08° ± 0.34° to 0.44° ± 0.47°. For BJ 1–4 ∑ 1–5 and CON 1–4 ∑ 1–5, the mean values were 0.32° ± 0.16° and 0.21° ± 0.25°, respectively and in BJ 1–4 ∑ 6–10 and CON 1–4 ∑ 6–10, the mean values were 0.36° ± 0.09° and 0.20° ± 0.22°, respectively.

## Discussion

This proof of concept was initiated to evaluate a measuring device capable of an optical measurement method for consistent comparison of the position stability of IACs with regard to its 3D-feasibility, accuracy, repeatability, time efficiency, and complexity.

Therefore, a comprehensive decision matrix was developed to systematically compare measuring devices. Through this process, the CMM was identified as the most suitable measuring device. Other devices, such as the Profile Projector, the DIC, the Profilometer, and the Confocal Technology, inadequately met the requirements defined in the decision matrix and where not further pursued. The CMM achieved 40/50 points in the decision matrix since it achieved accurate and repeatable results and is adequately time efficient, although it can be considered a medium complex device. Time efficiency and complexity could be improved through automation of locating the measuring points. With this adjustment, the complexity could be classified as simple (simple = 5 instead of medium = 3 points) and thus enhancing the total score. The Profile Projector only scored 30/50 points and was excluded at the beginning of the project because of feasibility limitations, inadequately visualizing the rotational deviation due to the 2D shadow image. Although the DIC is an accurate, medium complex and time-efficient method, we had to dismiss it as it needs individual calibration for every measurement with varying accuracies and this proof of concept was intended to serve as basis for further and repeatable IAC testing. The Profilometer and Confocal Technology scored 34/50 and 38/50 points, were in good midfield of the range from 10 to 50 points and thus promising devices. The Profilometer however showed a field of view that was too small in micro mode, which was required to achieve accurate results. In addition, the titanium surface was reflective, which negatively affected the interpretation, while the Confocal Technology is a difficult device and measurement implementation time consuming (> 1.5 h).

This study encompasses a dedicated and structured process of established measuring devices and the development of a complex decision matrix, which the CMM confirmed to us as sufficient to evaluate the vertical deviation and rotation in IACs. It may also help to find a suitable device for individual requirements. Since this proof of concept was intended as a pilot study, the small number of different IACs can certainly be discussed, however, based on these findings further IACs will be examined using this method. We do not declare completeness of the measuring devices and do not exclude the option that other measuring devices could be included in the decision matrix, since only accessible devices were evaluated. Further limitations of the study were the manual process of measurement and data evaluation. Automation could hypothetically improve accuracy, repeatability and efficiency. Additionally, the individual positioning of the measuring points of the CMM potentially introduce human error.

To contextualize our results within the existing literature, several related studies were noteworthy. To the best of our knowledge, no study has utilized a Profilometer nor a Profile Projector to measure IACs, even though these devices initially seemed viable options. Moreover, while these measuring machines have not been documented for evaluating IACs, the literature does not seem to explore the reasons these tools may be less suitable—a perspective we aim to provide through our decision matrix. They are mostly employed in mechanical testing, load application, and the evaluation of surface treatments, such as those on dental implants and are also commonly used for assessing the accuracy of dental impressions [16, 20, 38]. Our findings align with the conclusion that Profilometers and Profile Projectors are not well-suited to measure the IAC. This limitation stem from challenges related the complexity of IAC geometries, or the inability of these devices to capture intricate internal features with sufficient precision [39].

Regarding the DIC a recent study by Jacobs et al. measured the displacement between abutment and implant after hand-tightening and applying a specific force in 3D onto conical implants. However, a measuring accuracy was not provided in the study, since the DIC was calibrated by capturing images of a 1-inch glass calibration grid to create a common world coordinate system, allowing the image positions from both cameras to be mapped to a unified 3D location. Mean displacement values and confidence intervals for the displacement in 3D were provided in box plots, but not numeric [15]. As evaluated in our decision matrix, DIC presented as a potential method for testing IACs, yet the calibration process introduces variability in both accuracy and repeatability.

Confocal technologies are not currently used to measure the 3D stability of IACs but e.g. are utilized to assess biofilm reduction on dental implants. With nanometer-level precision, these technologies are useful for evaluating antimicrobial nanoparticles and their effects. Our decision matrix suggests that confocal microscopy could also be applicable for measuring IACs, but the high accuracy would yield results that are clinically insignificant, and the time required for measurements would likely make this approach impractical [40].

The majority of studies reading the 3D positional stability of IACs have utilized optical CMMs to assess IACs [12, 41]. Semper et al. evaluated the integrity of IACs in dental implants using a tactile method with a CMM [4, 5, 11, 28]. It provided accurate and valuable results, that are still of interest and which we were able to confirm with this proof of concept. While other studies have investigated IACs, the heterogeneity of experimental setups limits the ability to draw direct comparisons [12]. In 2021, Bedouin et al. investigated the rotational freedom of IACs using a contactless measurement microscope (Inexiv VMA-2520; Nikon Metrology Inc.). Their findings demonstrated high accuracy, to the extent that no clinically significant consequences were observed. However, the study omitted information on the time efficiency and complexity of the measurement process, failing to address the trade-off between high accuracy and the potentially time-consuming or complex nature of the measurements [41].

The use of optical CMMs is increasing, though both tactile and optical CMMs have distinct advantages and limitations. Tactile measurements are influenced by material properties such as temperature, stiffness, and surface roughness, while optical measurements can be affected by sensor sensitivity, lens focal length, image brightness, and illumination. Despite autofocus mitigating some issues, other factors like ambient conditions and vibrations can still impact accuracy. A recent study found optical probes can yield similar results to tactile CMMs when measuring hole plates, though discrepancies may occur under certain conditions [42]. In this proof of concept, measurements were conducted using an optical system with a field of view of 600 × 600 × 350 mm. The CMM was placed in a controlled environment at a constant temperature of 20 °C to minimize thermal variation. To reduce the impact of vibrations, the CMM was positioned on a vibration-damping table. Autofocus was employed for each measurement point to standardize the magnification and lighting conditions, ensuring consistency and minimizing potential sources of error.

These studies are vital for gaining a deeper understanding of the 3D behavior of IACs. However, there is considerable heterogeneity in the measurement machines used across different studies, and the rationale behind choosing specific machines is often not provided. While CMMs have become a common choice, the literature is deficient in consistent explanations as to why a particular measurement machine is selected for these studies. This absence of clarification makes it difficult to assess the potential impact of machine selection on the outcomes and reliability of measurements, leaving a gap in the transparency of research methodologies.

To confirm the CMM as a valuable option, pervious data were verified in this proof of concept. When examining the vertical deviation and rotation of the BJ and CON geometries, which were secured using screw-tightening by hand or torque ratchet, distinct differences were observed between geometries with the CMM. The BJ connections exhibited a vertical deviation of less than 10 microns independent of the torque applied to the abutment screw. In contrast, the conical IAC were more affected as previously described [4, 5, 11, 28]. Based on these findings, the null hypothesis was rejected. Since each IAC was hand-tightened five times, a measurable trend emerges, indicating that the abutment was less likely to be forced into the conus. This could be attributed to titanium particles being rubbed off during the insertion and removal of the abutment [43]. However, as higher screw-tightening forces were applied, the abutment was pulled into the conus, reaching a vertical deviation compared to the initial position that might be clinically relevant and may influence the fit of the implant-supported prosthetic restoration. One should consider the variability in force application by different operators in a real world scenario during hand tightening, reflecting routine practice of dental technicians who adjust occlusion in the laboratory without applying torque. The rotational deviation remained small (less than 1.35°) across all measurements, confirming that an internal cam groove connection offers minimal rotational freedom or rather a high precision in fit, this is in line with previous investigations [4, 5, 7, 11, 12, 28, 41].

## Conclusion

In summary, we validated the CMM a suitable and applicable device to measure the 3D positional stability of IACs with a complex decision matrix, we established a structured experimental setup, verified previous data and were able to present a clinically relevant variance between butt joint and conical connections in vertical deviation, while rotational deviation was not influenced. Therefore, we demonstrated that these tests are clinically relevant and that variations in 3D positional stability can be expected across different geometries and manufacturers.

## Data Availability

The datasets used and/or analysed during the current study are available from the corresponding author on reasonable request.

## Abbreviations

IAC: Implant abutment connection
CMM: Coordinate measuring machine
DIC: Digital Image Correlation
BJ: Butt-joint
CON: Conical
